# Early-Stage Defense Mechanism of the Cotton Aphid *Aphis gossypii* Against Infection With the Insect-Killing Fungus *Beauveria bassiana* JEF-544

**DOI:** 10.3389/fimmu.2022.907088

**Published:** 2022-06-02

**Authors:** Yeram Im, So-Eun Park, Sue Yeon Lee, Jong-Cheol Kim, Jae Su Kim

**Affiliations:** ^1^ Department of Agricultural Biology, College of Agriculture and Life Sciences, Jeonbuk National University, Jeonju, South Korea; ^2^ Department of Agricultural Convergence Technology, Jeonbuk National University, Jeonju, South Korea

**Keywords:** *Aphis gossypii*, cotton aphid, *Beauveria bassiana*, transcriptome, ecdysone

## Abstract

*Aphis gossypii*, commonly known as the cotton aphid, is a widely distributed pest of agricultural crops and acts as a vector for many serious plant viruses. Cotton aphid shows high resistance to chemical insecticides due to rapid rates of genetic diversity as a result of its short life cycle, seasonal migration, and host alteration. As an alternative, entomopathogenic fungi can be used to control cotton aphids in an environmentally sound manner. However, little is known about how cotton aphids respond to fungal infection. In this work, a new *Beauveria bassiana* strain JEF-544 (*Bb* JEF-544) was selected and isolated through bioassays with high virulence against cotton aphid. Early response of cotton aphid to *Bb* JEF-544 infection was analyzed at the transcriptome level. Infected aphids were collected two days after treatment at 25% lethal time (LT_25_), and total RNA of non-infected and *Bb* JEF-544-infected aphids was independently subjected to sequencing. Infected aphids showed significant up-regulation of the insect hormone biosynthesis pathway. Bursicon (Burs) and crustacean cardioactive peptide (CCAP) receptors involved in molting along with ecdysone synthesis were also strongly up-regulated in the aphid response to the fungal infection. In the immune response, melanization in the hemocoel was significantly up-regulated, while phagocytosis was less actively transcribed. In conclusion, cotton aphids protect themselves from *Bb* JEF-544 infection by activating the immune response including melanization and insect molting hormones to shed infected cuticles. In addition to describing the initial stages of *Bb* JEF-544 infection at the transcriptome level, this work provides potential treatment targets and insight into how fungal isolates can effectively be used to control this serious aphid species.

## Introduction

*Aphis gossypii*, commonly known as the cotton aphid, is distributed worldwide and is a serious pest that causes damage to agricultural crop production. Cotton aphid sucks sap from the leaves, inflorescences, and stems, resulting in stunted plant growth, depletion of plant nutrient resources, and visible feeding damage (1). The cotton aphid is a vector for more than 50 plant viruses including potato virus, citrus tristeza virus, cucumber mosaic virus, and turnip mosaic virus (2–4). The host range for the cotton aphid includes more than 50 plant families including *Asteraceae*, *Cucurbitaceae*, *Rosaceae*, and *Solanaceae* (5–7). Cotton aphids reproduce *via* a viviparous pattern and overwinter as eggs at low temperatures (8, 9). Aphids can reproduce at high density through parthenogenesis in spring and summer and produce alate progeny (10), which can migrate to a second host and develop genetic diversity through sexual reproduction, which is a big challenge for pest management (11).

Chemical pesticides such as bifenthrin, deltamethrin, imidacloprid, and malathion have been used to control cotton aphids; however, prolonged exposure to chemicals increases insensitivity and chemical resistance through genetic modification at the target site (12–16). Acephate, which targets the aphid nervous system, lowers acetylcholinesterase (AChE) activity and increases cytochrome P450 monooxygenase detoxification. Resistance to acephate has been identified in aphid AChE (17). It was also reported that aphids exposed to imidacloprid developed cross-resistance to fenvalerate (14). Therefore, there is a limit to controlling cotton aphids with only chemical agents.

Entomopathogenic fungi are pathogenic to various pests and can be used as biological control agents by alternatively replacing chemical pesticides for cotton aphid management (18–20). The conidia of entomopathogenic fungi invade the aphid by attaching to the epidermis (21, 22). Entomopathogenic fungi kill insects by secreting secondary metabolites that act as toxins. *Beauveria bassiana* species are known to secrete beauvericin, bassianin, bassianolide, and oosporein after invading insects (23). Of the fungal species, *B. bassiana* and *Metharizium anisopliae* exhibit significantly high virulence against *Aphis gossypii* (24). *B. bassiana* is also pathogenic to other aphids including *Aphis craccivora*, *Sitobion avenae*, *Schizaphis graminum*, *Rhopalosiphum padi*, *Brevicoryne brassicae*, and *Lipaphis erysimi* (25–27). RNA sequencing showed that *Conidiobolus obscurus*, an aphid pathogenic fungus, overexpresses the cytolytic-like δ-endotoxin gene and serine proteases while invading and killing aphids (28). In addition, *Lecanicillium lecanii* is known to increase pathogenicity against aphids by producing an enzyme that hydrolyzes aphid chitin through Vlchit1 expression (29). However, few studies have investigated the aphid response to fungal infections to understand the molecular basis of fungal pathogenesis and to identify highly virulent entomopathogenic fungi for use in controlling aphids.

In this study, we isolated a highly virulent *B. bassiana* strain JEF-544 (*Bb* JEF-544) that has potential to control cotton aphids and to investigate the defense response of cotton aphids against this fungal pathogen. The Illumina sequencing platform was used to analyze the aphid transcriptome at the early stages of *Bb* JEF-544 infection. Differentially expressed genes (DEGs), gene ontology (GO), and enrichment analysis were performed on infected cotton aphids, and the results were analyzed to identify the initial defense mechanisms. This study will help determine the response of cotton aphids to fungal pathogen infection.

## Materials and Methods

### Insects

An *Aphis gossypii* cotton aphid colony was provided by the National Institute of Agricultural Science in Korea (https://www.rda.go.kr/). Cotton aphids were reared on third leaf stage cucumber plants (Ilmi Samcheok, Green Heart Bio, Yeoju, Korea) under laboratory conditions in acrylic cages at 26 ± 1°C, 50 ± 5% relative humidity (RH), and 16 h-light (L):8 h-dark (D) photoperiod. All experiments were conducted with wingless aphids.

### Fungal Isolates

*Beauveria bassiana* isolates were obtained from soil in Korea using a *Tenebrio molitor* baiting method (30). Genus and species were identified by sequencing with primers targeting the internal transcribed spacer sequences of fungal genomic DNA. The fungal isolates were stocked in 20% glycerin at -80°C at the Jeonbuk National University Entomopathogenic Fungal Platform (JEF-library) until used in experiments. A total of 99 *B. bassiana* isolates was used for selection of highly virulent fungi as potential cotton aphid control agents.

### Bioassay

Each *B. bassiana* isolate was cultured on 1/4 Sabouraud dextrose agar (SDA, BD Difco, USA) medium for 10 days in darkness at 25°C. The fungal conidia of each *B. bassiana* isolate was suspended in 0.03% siloxane solution (Silwet, FarmHanong Inc., Nonsan, Korea) at 1 ×10^7^ conidia/ml. A 1.0 ml aliquot of fungal conidial suspension was sprayed on cucumber leaf discs (110 mm diameter) and dried at room temperature for 1 h. Filter paper (No.2 Ø110, ADVANTEC, Tokyo, Japan) moistened with 500 μl distilled water was laid on a 90 mm Petri-dish (SPL Life Sciences, Pocheon, Korea) onto which the sprayed cucumber leaf disc was placed and infested with cotton aphid nymphs (about 52 aphids/leaf disc). All Petri-dishes were sealed and maintained under 26 ± 1°C and 16L:8D conditions, and the numbers of live and dead aphids were observed daily. A 0.03% siloxane solution was used as a negative control. In the first screening step, each treatment had only one replicate; in the following bioassays, three replicates were conducted for each treatment.

### RNA Extraction and cDNA Library Construction

Total RNA was extracted from *Bb* JEF-544-infected and non-infected aphids for transcriptome analysis. The treatment of cotton aphids with *Bb* JEF-544 followed the bioassay method described above. Samples for RNA extraction contained live nymphs on the second day (LT_25_) after treatment. Non-infected aphids were also collected as a control. The collected aphids (50 mg) were placed in a 1.5 ml microfuge tube with 1 ml of TRIzol reagent (Molecular Research Center Inc., Cincinnati, OH, USA). Total RNA was extracted with one repetition according to manufacturer instructions. Briefly, aphids in TRIzol reagent were homogenized using a plastic pestle for 2 min, followed by addition of 200 μl chloroform (Sigma-Aldrich, MO, US) and incubation for 5 min at room temperature for complete dissociation. Homogenates were centrifuged at 12,000 g for 15 min at 4°C, and 400 μl of the upper aqueous phase was transferred to a fresh tube with 400 μl 2-propanol (EMPARTA^®^, EMD Millipore, Darmstadt, Germany). Samples were incubated for 5 min at room temperature and centrifuged at 12,000 g for 10 min at 4°C. The supernatant was removed, and the RNA pellet was washed by vertexing with 75% absolute ethanol (Daejung, Siheung, Korea) and centrifuged at 7,500 g for 5 min at 4°C. The washed pellet was air-dried for 5 min and dissolved in RNAse-free water (UltraPure distilled water, Invitrogen, MA, US). Extracted RNA quality was verified by electrophoresis with 0.8% agarose gels, and quantity was measured using spectrophotometry (ASP-2680, ACTGene, NJ, USA). Sequencing libraries of *Bb* JEF-544-infected and non-infected aphids were constructed at Macrogen using the TruSeq Stranded Total RNA LT Sample Prep kit (Illumina, San Diego, CA, USA) according to manufacturer protocols. The prepared library was sequenced with a read length of 101 bp using the Illumina platform (NovaSeq 6000, Illumina, San Diego, CA, USA).

### Mapping and Differentially Expressed Gene Analysis

Obtained short reads were quality checked using FastQC (31) and trimmed using fastp preprocessing (32). To obtain the transcript per million (TPM) value, the short reads of non-infected and *Bb* JEF-544-infected aphids were mapped using the cotton aphid reference genome (GCF_004010815.1_ASM401081v1_rna, Aphidbase) using the kallisto program (33). The fold change (FC) value was obtained by dividing the TPM of *Bb* JEF-544-infected aphids by the TPM of non-infected aphids. Genes with TPM values less than 1 for both non-infected and infected aphids were removed, and those with a log_2_FC greater than 1 were used for DEG analysis.

### Validation for RNA-seq Analysis

For validation of RNA-sequencing, 10 DEGs were randomly selected (**Table S1**), and primers were designed using Primer3Plus (https://www.bioinformatics.nl/cgi-bin/primer3plus/primer3plus.cgi). Elongation factor 1 alpha (EF1α) was used as the internal control with the following primer sets: EF1α -F: GAAGCCTGGTATGGTTGTCGT and EF1α -R: GGGTGGGTTGTTCTTTGTG (34). cDNAs were synthesized with infected and non-infected aphid RNAs using AccuPower RT PreMix (Bioneer, Daejeon, Korea) with oligo (dT) 15 primer (Promega, MI, USA) according to manufacturer protocols. qRT-PCR was performed using Thunderbird SYBR qPCR mix (QPS-201, TOYOBO, Japan) and the CFX96 Touch Real-Time PCR Detection System (Bio-Rad, Hercules, CA, USA). PCR was conducted under the following conditions: 95°C for 1 min, followed by 40 cycles of 95°C for 15 s and 60°C for 1 min. At the end of each PCR run, a melting curve from 65°C to 95°C increased by 0.5°C per 5 s was applied to ensure the specificity of the amplicon. All experiments were performed in triplicate, and the FC value was obtained using the 2^-ΔΔCt^ method.

### Analysis of Functional Changes in Cotton Aphid Transcriptomes

For gene ontology (GO) analysis, the DEGs of *Bb* JEF-544-infected and non-infected cotton aphids were entered into the EMBL-EBI database (https://www.ebi.ac.uk/) and analyzed using the Blast2Go program with InterProScan and annotated with GO identifiers (IDs) and GO terms. The Ensembl genomes database was used for functional profiling of up- and down-regulated genes in *Bb* JEF-544-infected aphids annotated to the pea aphid reference (aphidbase_2.1b_transcripts.fasta) using BLASTN. The annotated up- and down-regulated genes were further analyzed using the enrichment program g:Profiler (https://biit.cs.ut.ee/gprofiler/gost) with reference to the *Acyrthosiphon pisum* (pea aphid) genome. A Benjamini-Hochberg false discovery rate (FDR) <0.05 was used as the threshold.

### Change in Expression of Insect Hormone and Immune-Related Genes

To analyze the expression levels of immune defense genes in *Bb* JEF-544-infected aphids, the sequences of insect hormone and immune genes were downloaded from Swiss-Prot and the TrEMBL database from UniProt (https://www.uniprot.org/), which includes the genes for the insect hormones ecdysone, bursicon, crustacean cardioactive peptide, phagocytosis, encapsulation, and melanization (**Table S5**). Insect hormone- and immune-related genes were converted into a BLAST database. Defense-related DEGs of the cotton aphid were identified with an E-value 1.0e^-100^ using Blast2Go from the local BLAST database.

### Statistical Analysis

Bioassay data were analyzed using one-way analysis of variance (ANOVA) using Tukey’s HSD for multiple comparison, and *p*<0.05 was considered statistically significant. The mortality data of *Bb* JEF-544-infected and non-infected cotton aphids were subjected to Probit analysis to calculate 25% lethal time. ANOVA and Probit analyses were conducted using SPSS Statistics 19 software (SPSS Inc., Chicago, IL, USA).

## Results

### Virulence of *Beauveria bassiana* JEF-544 Against Cotton Aphid

**Figure 1:**
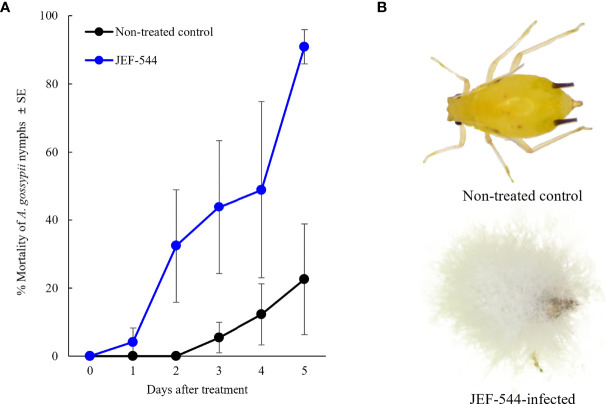
Virulence of *B bassiana* JEF-544 against cotton aphid in laboratory conditions. **(A)** Mortality of cotton aphids against *B bassiana* JEF-544 (F_1,11 =_ 7.173, *p* = 0.12) and **(B)** Non-infected and JEF-544-infected aphid nymphs 6 days after treatment.

Of 99 *B. bassiana* isolates against cotton aphid, bioassays revealed 12 that showed high virulence of 80% or greater mortality within 5 days after treatment (**Figure S1**). In a second bioassay, the *Bb* JEF-544 isolate demonstrated greater than 90% mortality against cotton aphids within 5 days after treatment (F_1,11 =_ 7.173, *p*=0.12; **Figure 1**). The LT_25_ of *Bb* JEF-544-infected aphids was confirmed as 2.27 days (range: 1.08-2.96 days) by Probit analysis (χ2 = 489.094, df=16, *p*<0.001). Infected cotton aphids turned dark 6 days after treatment. In the aphid cadavers, the entire body was covered with white mycelium due to mycosis, and conidia formed on top of the mycelial mass.

### Differentially Expressed Cotton Aphid Genes due to *Beauveria bassiana* JEF-544 Infection

**Figure 2:**
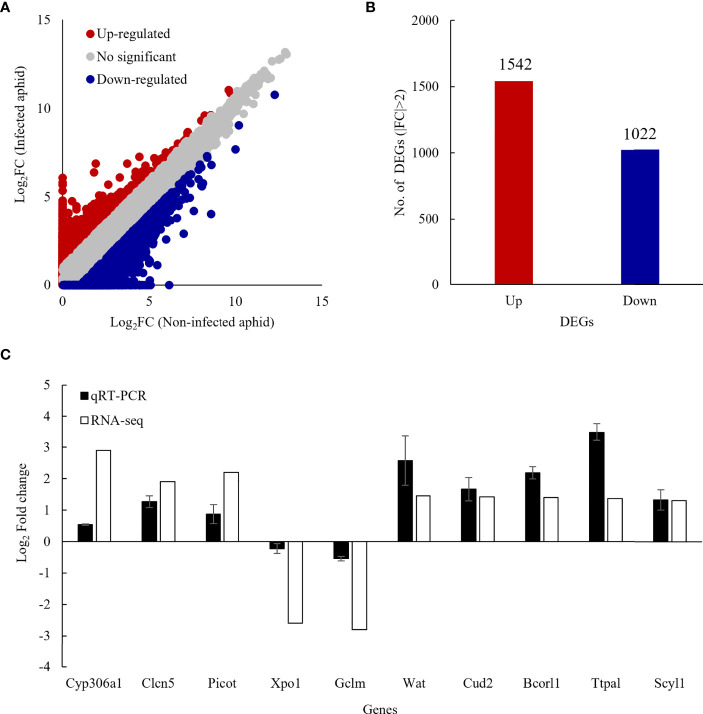
Differentially expressed genes (DEGs) of infected coon aphids. **(A)** Scatter plot comparing log2 ratios of TPM expression values of non-infected and JEF-544-infected aphids, **(B)** Number of DEGs in JEF-544-infected aphids (|FC|>2), **(C)** Validation of RNA-seq analysis using qRT-PCR. CYP306a1, cytochrome P450 306a1; Clen5, H(+)/Cl (–) exchange transporter 5; Picot, putative inorganic phosphate cotransporter; Xpo1, exportin-1; Gclm, glutamate-cysteine ligase regulatory subunit; Wat, fatty acyl-CoA reductase wat; Cud2, endocuticle structural glycoprotein SgAbd-2; Bcorl1, BCL-6 corepressor-like protein 1; Ttpal, alpha-tocopherol transfer protein; Scyl1, N-terminal kinase.

The RNA sequence produced 71,589,088 raw reads in the non-infected and 68,792,464 raw reads in the *Bb* JEF-544-infected cotton aphids. The data were submitted to NCBI under the accession number PRJNA815895 (**Table S2**). Short reads were mapped to the reference genome after filtering. In both *Bb* JEF-544-infected and non-infected cotton aphids, the distribution of DEGs was confirmed based on log_2_FC (FC>1) except for genes having a TPM value less than 1 (**Figure 2A**). Of the DEGs, 1,542 were up-regulated and 1,022 were down-regulated in the *Bb* JEF-544-infected aphid samples (**Figure 2B**), and a relatively large number of genes was up-regulated (log_2_FC>1). Validation was performed through qRT-PCR using EF1α as an internal control for randomly selected genes. The results of the RNA-seq analysis of 10 genes and the gene expression patterns of qRT-PCR were similar (**Figure 2C**).

### Functions of DEGs of Infected Cotton Aphids

**Figure 3:**
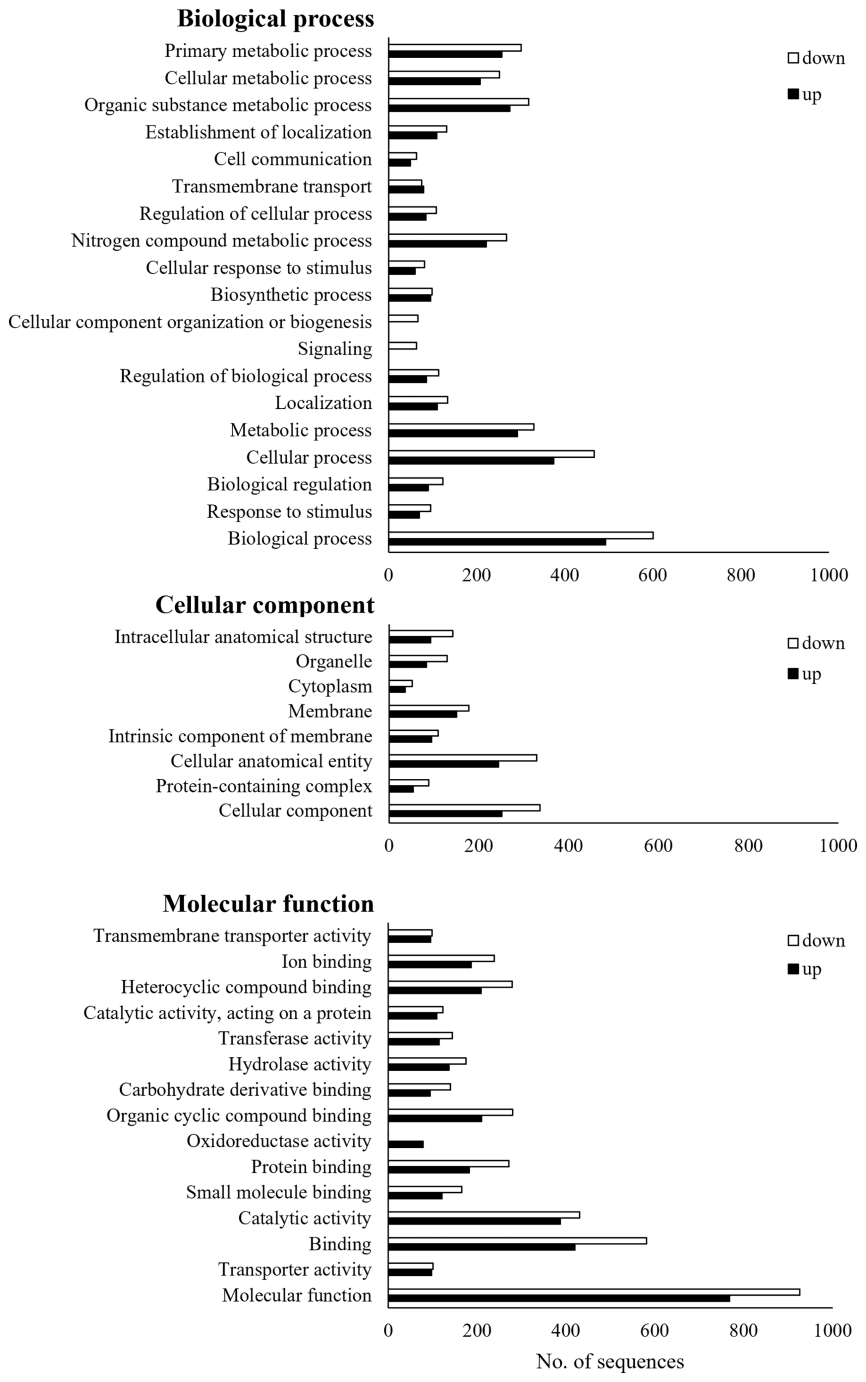
Gene ontology (GO) analysis of JEF-544-infected aphid. DEGs of JEF-544-infected aphids were classified into three main categories (biological process, cellular component, and molecular function).

**Figure 4:**
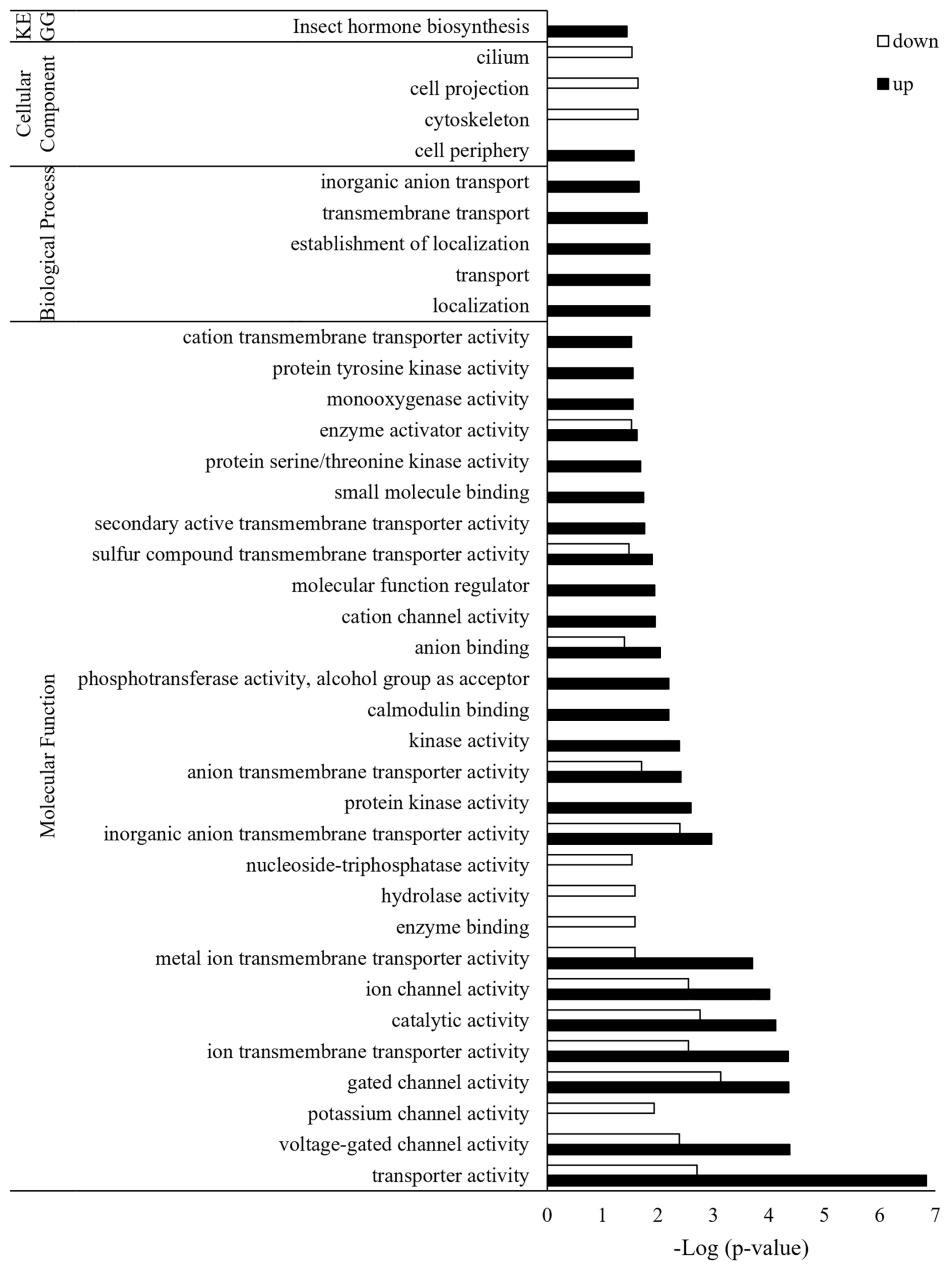
GO enrichment analysis of JEF-544-infected aphid genes. Enrichment analysis was performed to confirm significant functions among DEGs of infected aphids. FDR <0.05 was used as the significance threshold, and *Acyrthosiphon pisum* was used as the reference.

Of the total 2,564 DEGs, 1,787 were annotated, and each was classified into GO of biological process, cellular components, and molecular function (**Figure 3**). Three GO terms were classified down to GO level 3. Up- and down-regulated genes were found together in most GO terms, but there were more down-regulated genes than up-regulated genes. The only up-regulated GO term identified in *Bb* JEF-544-infected aphids was oxidoreductase activity (GO:0016491). The only down-regulated GO terms, which were found only in the *Bb* JEF-544-infected aphids, were signaling (GO:0023052) and cellular component organization or biogenesis (GO:0071840). From the enrichment analysis of up- and down-regulated genes using g:Profiler, the up-regulated genes were enriched in 115 GO terms and in one KEGG pathway (**Table S3**). In addition, down-regulated genes were enriched in 34 GO terms (**Table S4**). A total of 2,155 genes was enriched to molecular function (76.71%), biological process (19.95%), cellular component (3.01%), or KEGG (0.32%). Transporter activity (GO:0005215), anion binding (GO:0043168), small-molecule binding (GO:0036094), and insect hormone biosynthesis (KEGG:00981) were up-regulated and showed negative log p-values of 6.83, 2.03, 1.74 and 1.43, respectively. A total of 615 genes was enriched to molecular function (92.52%) or cellular component (7.48%). Catalytic activity (GO:0005215), hydrolase activity (GO:0016787), and cytoskeleton (GO:0005856) were down-regulated and showed negative log *p*-values of 2.76, 1.58, and 1.63, respectively (**Figure 4**).

### Molting Hormone-Mediated Response Against *Beauveria bassiana* JEF-544 Infection

**Figure 5:**
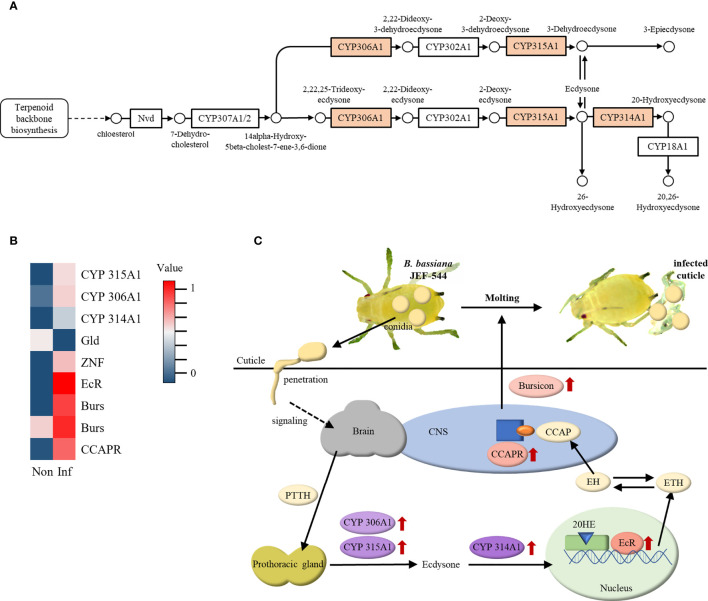
Expression of insect hormone-related genes. **(A)** Enriched insect hormone biosynthesis pathway (analyzed by g:Profiler), **(B)** Heatmap of insect hormone-related genes with expression level, and **(C)** Molting response in JEF-544-infected aphid as a defense behavior.

The insect hormone biosynthesis pathway was highly expressed in *Bb* JEF-544-infected cotton aphids at LT_25_ of treatment, and genes for cytochrome P450 were significantly up-regulated (**Figure 5A**). Cytochrome P450 (CYP) 306A1, 315A1, and 314A1 are involved in the molting hormone synthesis pathway. CYP306A1 and CYP314A1 (ecdysone 20-monooxyganase) are genes that synthesize ecdysone and 20-hydroxyecdysone (20HE), respectively, which are essential for molting. As a result of the expression level of insect hormone-related genes, ecdysone receptor (EcR), bursicon (Burs), crustacean cardioactive peptide (CCAP) receptor, and zinc finger protein (ZNF) were significantly up-regulated in *Bb* JEF-544-infected cotton aphids (**Figure 5B**). Based on the enrichment analysis and heatmap, the ecdysis response of *Bb* JEF-544-infected cotton aphids is presented in **Figure 5C**.

### Cotton Aphid Immune Response Against *Beauveria bassiana* JEF-544 Infection

**Figure 6:**
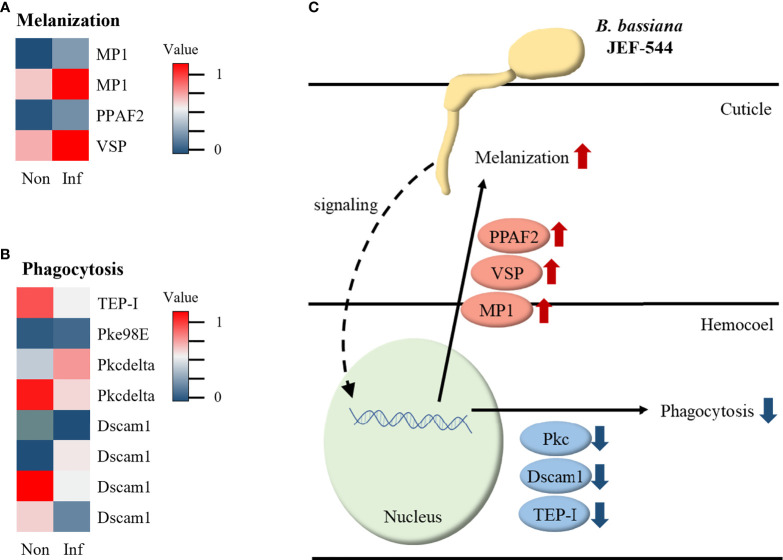
Expression of immune-related genes in infected aphid. **(A)** Heatmap of melanization-related genes with expression level, **(B)** Heatmap of phagocytosis-related genes with expression level and **(C)** Summary of immune response in JEF-544-infected aphid.

From the analyses of cotton aphid DEGs in terms of immunity, several genes were identified relative to melanization and phagocytosis *via* BLAST analysis. During melanization, melanization protease 1 (MP1), phenoloxidase-activating factor 2 (PPAF2), and venom serine protease (VSP), which activate phenol oxidase, were up-regulated (**Figure 6A**). Of the genes involved in phagocytosis, protein kinase C (PKC98E) and kinase C delta type homolog (PKCdelta) were up-regulated, whereas thioester-containing protein I (TEP-I), PKCdelta, and most isoforms of down syndrome cell adhesion molecule 1 (Dscam1) were down-regulated (**Figure 6B**). According to the heatmap data, cotton aphid melanization and phagocytosis response against *Bb* JEF-544 are presented in **Figure 6C**.

## Discussion

Cotton aphid is a serious pest, and control using entomopathogenic fungi has recently attracted attention. However, the defense mechanism of cotton aphids against invasion of entomopathogenic fungi is not well understood. This study analyzed the transcriptional response of cotton aphid when infected with *Beauveria bassiana* JEF-544, a newly-isolated and highly virulent fungal strain. The immune system of insects evolved to remove pathogens during the early stages of infection. Therefore, we confirmed the active defense response of the cotton aphid at LT_25_, which is an early stage of infection (35). The cotton aphid rapidly uncovered fungus-treated cuticles, and melanization occurred at LT_25_, the initial stage of infection.

For selection of candidate entomopathogenic control agents for cotton aphids, 99 *B. bassiana* strains were isolated using an insect baiting method and evaluated for insecticidal activity against aphids. In the bioassay, *Bb* JEF-544 showed the fastest and highest insecticidal activity among the 99 candidate isolates. *Bb* JEF-544 provided strong control over growing aphid populations, limiting growth to an average of 2.8 offspring per adult (36). It is expected that *Bb* JEF-544 is a good candidate for biological control against cotton aphids. Mass production, formulation, and field experiments will be necessary to establish this strain as an entomopathogenic control agent.

Cotton aphid transcripts were generated two days after infection (LT_25_) to analyze the early response of cotton aphids against *Bb* JEF-544 infection. To kill the host, entomopathogenic fungi including *B. bassiana* go through the stages of attachment, penetration, fungal growth in hemolymph, conidia production, and transmission (22). The live aphids 2 days after treatment, which was LT_25_ of *Bb* JEF-544, were considered to be in the early stage of infection, and mostly fungal conidia were presumed to be working on penetrating aphid cuticles. Insects defend themselves by modifying the cuticle as a physical barrier and innate immune response when pathogens invade (37). Pathogens are recognized inside the insect body and activate signaling pathways such as Toll, immune deficiency (IMD), Jun N-terminal kinase (JNK), and prophenoloxidase (PPO), and the consequent immune response occurs through production of antimicrobial peptide (AMP) and the cellular immune response. In a previous study, genes of the immune-related Toll and IMD pathways were up-regulated during infection by the Japanese pine sawyer beetle, *Monochamus alternatus*, which was infected with the fungal pathogen *M. anisopliae* (38). Longhorned ticks in the early stages of *M. anisopliae* infection expend a large amount of energy in catabolic processes against the fungal attack (39). The citrus whitefly *Dialeurodes citri*, when infected with *Lecanicillium attenuatum*, up-regulated genes for vitellogenin, PPO, and lysozyme (40). However, contrary to other known insects, aphids do not encode genes for PGRP, IMD, AMP, and other immune-related molecules (41, 42). Aphids, which have an incomplete immune system, recognize bacterial pathogens *via* the JNK pathway, and the immune response occurs through hemocyte-mediated responses and phenoloxidase (PO) (43). Likewise, Toll- and IMD-related genes were not identified, and in this study, insect hormone biosynthetic pathways and melanogenesis activators were up-regulated in the early stages of infection.

Ecdysone is one of the molting hormones in insects, along with various factors such as prothoracicotropic hormone (PTTH), ecdysis-triggering hormone (ETH), eclosion hormone (EH), crustacean cardioactive peptide (CCAP) and bursicon (44–46). PTTH secreted from the brain stimulates prothoracic gland (PG), and CYP306A1 and 315A1 synthesize ecdysone (47). When ecdysone is oxidized by CYP314A1, it becomes 20-hydroxyecdysone (20HE), an activated form. 20HE acts as a transcription factor by binding to the ecdysone receptor (EcR) in the nucleus and secretes ETH (48–50). ETH increases the release of EH as a positive feedback, and EH stimulates the secretion of CCAP (51). CCAP inhibits the pre-ecdysis response and stimulates the molting process (52). CCAP binds to CCAPR in the central nervous system (CNS) and stimulates bursicon secretion. Bursicon then functions to tan the cuticle and induces post-ecdysis behavior. In this study, ecdysone, 20HE, CCAP receptor, and Burs were up-regulated in cotton aphids at early stages of *Bb* JEF-544 infection. This might be the aphid response to shed the infected cuticle and remove the initial infecting fungal mass invading through the cuticle. The expression levels of Burs and CCAP receptor were significantly increased relative to that of CYP. It is inferred that the molting process in cotton aphids entered the latter stage at LT_25_ after fungal pathogen invasion.

PPO can activate PO to decrease the number of invading *B. bassiana* spores in aphids (53). PPO is activated by VSP, PPAF, and MP1 and induces melanogenesis (54–56). In this study, the MP1, PPAF2, and VSP genes that activate the PO cascade were overexpressed. From this, it is inferred that the melanization reaction can occur in cotton aphids when infected by *Bb* JEF-544. Phagocytosis is a very rapid reaction against invading pathogens (52, 53). In *Bb* JEF-544-infected aphids, some genes including thioester-containing protein I (TEP-I), PKCdelta, and Dscam1, which are involved in phagocytosis, were identified (57). However, most genes tended to be down-regulated, and it is inferred that the phagocytosis response did not occur through these down-regulated genes. Melanization is a reaction that occurs in the endocuticle of insects (58). However, phagocytosis occurs in the hemocoel by hemocytes and is a very rapid response that occurs within 5 minutes of pathogen invasion (59, 60). This study was analyzed on LT_25_ of aphids, and it was expected to see the response in the early stages of infection. According to the down-regulation of phagocytosis-related genes, it is inferred that the fungus has not yet reached the hemocoel of aphids.

However, *B. bassiana* BCC2660 could form hyphal bodies in green peach aphid, *Myzus persicae* 3 days after treatment (61). This means that *B. bassiana* can invade the aphid body rapidly after treatment. The entomopathogenic fungi *B. bassiana* and *M. anisopliae* could evade and survive against the phagocytic hemocyte cells of the insect hemolymph (62). The down-regulation of phagocytosis-related genes speculates that *B. bassiana* JEF-544 evades the immune response that occurs in the hemocoel of aphids and proliferates. A further study of phagocytes in the infected aphids is required.

Cotton aphids responded to the *Bb* JEF-544 infection by molting to uncover the infected cuticles at the initial stage of fungal infection. In the hemocoel, the immune response to invading fungal pathogen was achieved through melanization, which was initiated by the PO cascade, but phagocytosis involving the hemocyte did not actively occur. This study is limited to LT_25_ after infection, and it is inferred that gene expression at different times after infection could confirm and/or identify the involvement of different reactions. Overall, this work is a strong platform to understand the cotton aphid response to early fungal infection and provides ideas for fungal isolates that could be effectively used to control this serious aphid species at the molecular level.

## Data Availability Statement

The datasets presented in this study can be found in online repositories. The names of the repository/repositories and accession number(s) can be found in the article/supplementary material.

## Author Contributions

YI, S-EP and SL carried out the experiment. YI and J-CK analyzed RNA sequencing raw data. YI and JK designed the experiments and wrote the manuscript. All authors contributed to the article and approved the submission of this manuscript.

## Funding

This work was supported by Korea Institute of Planning and Evaluation for Technology in Food, Agriculture and Forestry (IPET) through Plant Virus and Industrialization in Response to Pests Program, funded by Ministry of Agriculture, Food and Rural Affairs (MAFRA) (Grant No: 120080-05) and for Educating Creative Global Leader Program (or Project), funded by Ministry of Agriculture, Food and Rural Affairs (MAFRA) (Grant No: 321001-03).

## Conflict of Interest

The authors declare that the research was conducted in the absence of any commercial or financial relationships that could be construed as a potential conflict of interest.

## Publisher’s Note

All claims expressed in this article are solely those of the authors and do not necessarily represent those of their affiliated organizations, or those of the publisher, the editors and the reviewers. Any product that may be evaluated in this article, or claim that may be made by its manufacturer, is not guaranteed or endorsed by the publisher.
