# Supplementary material for: Early-Stage Defense Mechanism of the Cotton Aphid Aphis gossypii Against Infection With the Insect-Killing Fungus Beauveria bassiana JEF-544
Source: Front Immunol. 2022 Jun 2;13:907088. doi: 10.3389/fimmu.2022.907088 (PMC9201107; doi:10.3389/fimmu.2022.907088)
Supplement: Supplementary file 1 [file Presentation_1.pptx]

## Slide 1
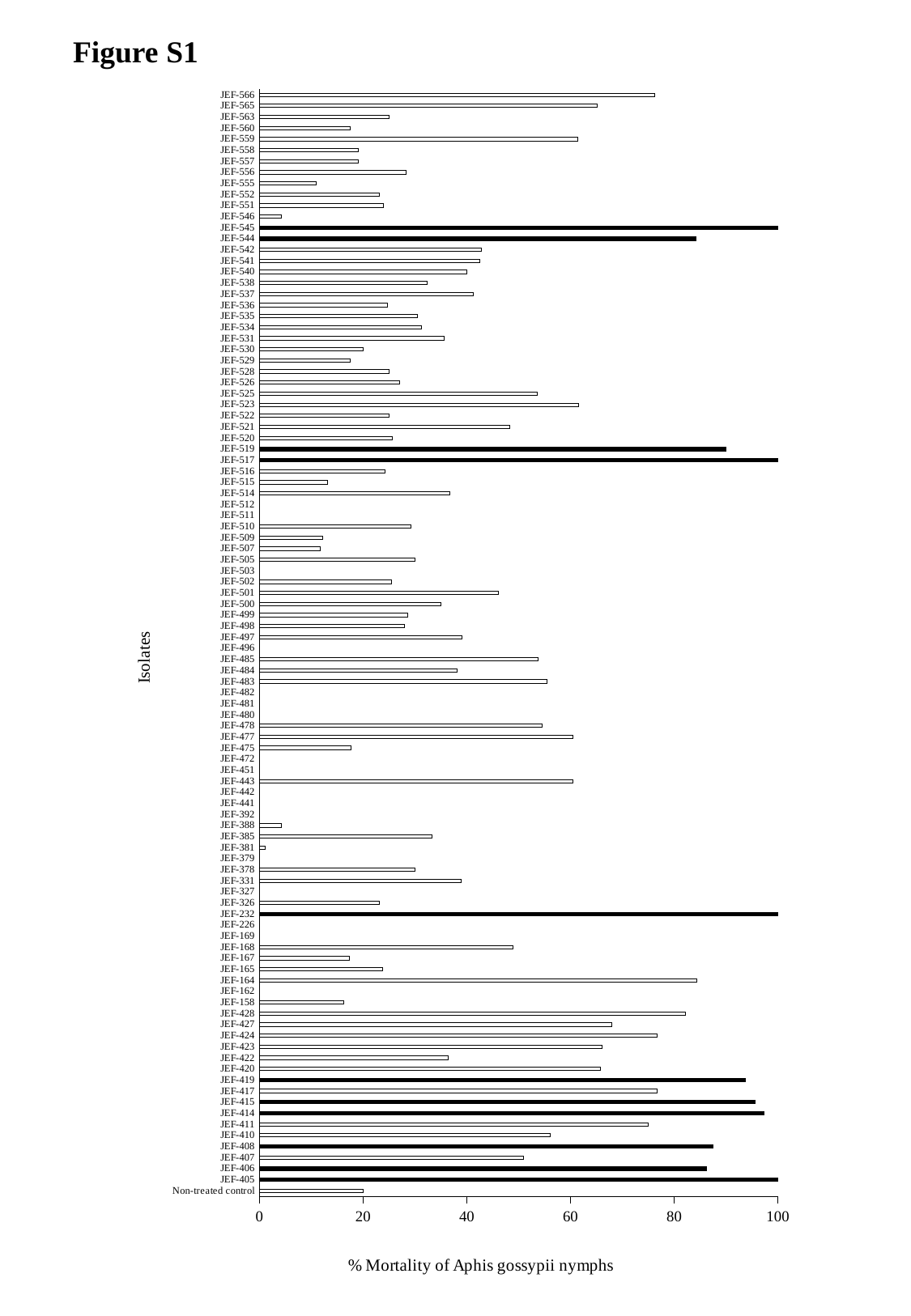

Figure S1
### Chart
| Category | |
|---|---|
| Non-treated control | 20.0 |
| JEF-405 | 100.0 |
| JEF-406 | 86.20689655172414 |
| JEF-407 | 50.943396226415096 |
| JEF-408 | 87.5 |
| JEF-410 | 56.16438356164384 |
| JEF-411 | 75.0 |
| JEF-414 | 97.29729729729729 |
| JEF-415 | 95.65217391304348 |
| JEF-417 | 76.66666666666667 |
| JEF-419 | 93.75 |
| JEF-420 | 65.78947368421052 |
| JEF-422 | 36.36363636363637 |
| JEF-423 | 66.10169491525424 |
| JEF-424 | 76.66666666666667 |
| JEF-427 | 67.94871794871796 |
| JEF-428 | 82.22222222222223 |
| JEF-158 | 16.21621621621621 |
| JEF-162 | -13.63636363636364 |
| JEF-164 | 84.41558441558442 |
| JEF-165 | 23.70370370370371 |
| JEF-167 | 17.391304347826093 |
| JEF-168 | 48.93617021276596 |
| JEF-169 | -74.41860465116278 |
| JEF-226 | -45.45454545454547 |
| JEF-232 | 100.0 |
| JEF-326 | 23.076923076923066 |
| JEF-327 | -56.0 |
| JEF-331 | 38.888888888888886 |
| JEF-378 | 30.075187969924812 |
| JEF-379 | -3.448275862068968 |
| JEF-381 | 1.1235955056179847 |
| JEF-385 | 33.33333333333334 |
| JEF-388 | 4.225352112676063 |
| JEF-392 | -66.03773584905662 |
| JEF-441 | 0.0 |
| JEF-442 | -12.658227848101262 |
| JEF-443 | 60.43956043956044 |
| JEF-451 | -82.5 |
| JEF-472 | -39.393939393939405 |
| JEF-475 | 17.64705882352942 |
| JEF-477 | 60.41666666666667 |
| JEF-478 | 54.54545454545455 |
| JEF-480 | -294.11764705882354 |
| JEF-481 | -27.397260273972606 |
| JEF-482 | -65.78947368421052 |
| JEF-483 | 55.55555555555556 |
| JEF-484 | 38.1578947368421 |
| JEF-485 | 53.75722543352601 |
| JEF-496 | -359.99999999999994 |
| JEF-497 | 39.032258064516135 |
| JEF-498 | 28.0 |
| JEF-499 | 28.57142857142857 |
| JEF-500 | 35.0 |
| JEF-501 | 46.15384615384615 |
| JEF-502 | 25.5 |
| JEF-503 | -322.49999999999994 |
| JEF-505 | 30.0 |
| JEF-507 | 11.666666666666671 |
| JEF-509 | 12.272727272727266 |
| JEF-510 | 29.285714285714278 |
| JEF-511 | -44.28571428571428 |
| JEF-512 | -154.99999999999997 |
| JEF-514 | 36.66666666666667 |
| JEF-515 | 13.07692307692308 |
| JEF-516 | 24.285714285714292 |
| JEF-517 | 100.0 |
| JEF-519 | 90.0 |
| JEF-520 | 25.66666666666667 |
| JEF-521 | 48.33333333333333 |
| JEF-522 | 25.0 |
| JEF-523 | 61.515151515151516 |
| JEF-525 | 53.57142857142857 |
| JEF-526 | 27.083333333333343 |
| JEF-528 | 25.0 |
| JEF-529 | 17.5 |
| JEF-530 | 20.0 |
| JEF-531 | 35.71428571428571 |
| JEF-534 | 31.33333333333333 |
| JEF-535 | 30.434782608695656 |
| JEF-536 | 24.64285714285714 |
| JEF-537 | 41.30434782608695 |
| JEF-538 | 32.38095238095238 |
| JEF-540 | 40.0 |
| JEF-541 | 42.50000000000001 |
| JEF-542 | 42.85714285714286 |
| JEF-544 | 84.21052631578948 |
| JEF-545 | 100.0 |
| JEF-546 | 4.166666666666657 |
| JEF-551 | 24.0 |
| JEF-552 | 23.181818181818187 |
| JEF-555 | 11.0 |
| JEF-556 | 28.33333333333333 |
| JEF-557 | 19.130434782608702 |
| JEF-558 | 19.090909090909093 |
| JEF-559 | 61.40350877192983 |
| JEF-560 | 17.5 |
| JEF-563 | 25.0 |
| JEF-565 | 65.11627906976744 |
| JEF-566 | 76.25 |
